# Dulaglutide versus empagliflozin as add-on therapy to metformin and sulfonylurea in type 2 diabetes: a randomized pilot study with exploratory metabolomic and microbiome analyses

**DOI:** 10.3389/fendo.2026.1843595

**Published:** 2026-06-17

**Authors:** Ji Eun Jun, Da-Hee Oh, In-Kyung Jeong, Hyun Jin Ryu, You-Cheol Hwang, Kyu Jeung Ahn, Ho Yeon Chung, Kwang Pyo Kim

**Affiliations:** 1 Division of Endocrinology and Metabolism, Department of Medicine, Kyung Hee University School of Medicine, Kyung Hee University Hospital at Gangdong, Seoul, Republic of Korea; 2 Department of Biomedical Science and Technology, Graduate School, Kyung Hee University, Seoul, Republic of Korea; 3 Department of Applied Chemistry, Institute of Natural Science, Kyung Hee University, Yongin, Republic of Korea

**Keywords:** GLP-1 receptor agonist, gut microbiota, metabolites, SGLT2 (sodium-glucose cotransporter 2) inhibitor, type 2 diabetes

## Abstract

In patients with type 2 diabetes mellitus (T2DM) inadequately controlled with metformin and sulfonylurea, evidence directly comparing glucagon-like peptide-1 receptor agonists and sodium–glucose cotransporter 2 inhibitors as add-on therapy is limited; therefore, we compared dulaglutide and empagliflozin in this setting. This 12-week, single-center, randomized, open-label, parallel-group pilot study included a 24-week observational extension. Patients with HbA1c≥7.0% receiving stable doses of metformin and glimepiride were randomized to dulaglutide 0.75 mg/week or empagliflozin 10 mg/day. Doses were uptitrated at week 4 if tolerated and maintained for 12 weeks, with follow-up until week 36. The primary endpoint was the change in HbA1c at week 12. Secondary endpoints included changes in glycemic and obesity-related parameters. Exploratory analyses were performed to assess plasma metabolite profiles using liquid chromatography-mass spectrometry, and gut microbiota using 16S rRNA gene sequencing. Twenty-four patients completed the 12-week study (dulaglutide, n=13; empagliflozin, n=11). Both treatments significantly reduced HbA1c at week 12, with no significant between-group difference. Empagliflozin significantly reduced HOMA-IR, whereas dulaglutide significantly increased HOMA-β. At week 12, empagliflozin was associated with greater reductions in body weight and body fat compared with dulaglutide, whereas these differences were attenuated at week 36. Exploratory analyses suggested potential, modest treatment-related differences in plasma metabolite profiles and microbiome–metabolic associations, without marked alterations in overall microbial diversity. As add-on therapy to metformin and sulfonylurea, both dulaglutide and empagliflozin improved glycemic control, with no significant between-group difference observed in this exploratory pilot study. Empagliflozin induced earlier weight loss, whereas dulaglutide showed more gradual weight reduction over time, accompanied by exploratory findings suggesting possible differences in plasma and microbiome-related metabolic signatures.

## Introduction

Glucagon-like peptide-1 receptor agonists (GLP-1RAs) and sodium–glucose cotransporter 2 inhibitors (SGLT2is) are effective therapies for improving glycemic control and promoting weight loss in patients with type 2 diabetes mellitus (T2DM) (1). GLP-1RAs mimic the action of endogenous GLP-1, thereby enhancing glucose-dependent insulin secretion, suppressing glucagon release, delaying gastric emptying, and reducing food intake through central appetite suppression (2). In contrast, SGLT2is inhibit renal glucose reabsorption in the proximal tubule, leading to glycosuria, caloric loss, and improvement of glucotoxicity (3).

Recent clinical guidelines have shifted toward an individualized treatment paradigm that prioritizes glycemic control and weight management, in addition to the presence of cardiovascular or renal disease (4, 5). From this perspective, both GLP-1RAs and SGLT2is are strongly recommended owing to their favorable effects on body weight and cardio-renal outcomes (4, 5). Given their distinct pharmacological mechanisms and organ targets (6), these agents are commonly considered as add-on therapies to conventional antidiabetic medications. Metformin combined with sulfonylurea remains one of the most frequently prescribed regimens for T2DM (7). When glycemic targets are not achieved with this combination, intensification with a third-line glucose-lowering agent is recommended (8); however, clear guidance is lacking regarding the optimal choice between GLP-1RAs and SGLT2is in this setting.

Beyond their glucose-lowering effects, emerging evidence suggests that bile acids and the gut microbiota may contribute to metabolic regulation through complex enterohepatic and hormonal signaling pathways. Bile acids are increasingly recognized as metabolic signaling molecules that influence glucose homeostasis (9), in part by stimulating GLP-1 secretion and interacting with nuclear receptors (10, 11). Experimental and mechanistic studies suggest that GLP-1RAs and SGLT2is may influence bile acid metabolism and circulating bile acid profiles (12, 13), although the clinical significance of these changes remains incompletely understood. In addition, the gut microbiota has been implicated in the regulation of host metabolism, insulin sensitivity, and energy balance (14). Alterations in gut microbial composition have been reported following treatment with GLP-1RAs or SGLT2is, potentially reflecting differences in microbial responses rather than profound community-wide restructuring (15). However, data from head-to-head clinical studies comparing these agents remain limited, particularly regarding their metabolic effects, with only limited exploratory data on bile acids and gut microbiota.

Therefore, this study aimed to compare the efficacy and safety of dulaglutide and empagliflozin as add-on therapy in patients with T2DM inadequately controlled with metformin and sulfonylurea. Exploratory analyses were additionally performed to assess potential changes in plasma metabolites and gut microbiota.

## Methods

### Study participants

Patients aged 20–75 years with T2DM who had been treated with a stable dose of metformin (≥1000 mg/day) and glimepiride (≥4 mg/day) for at least 8 weeks were eligible for enrollment. Inclusion required a glycated hemoglobin A1c (HbA1c) level of ≥7.0% at screening. Key exclusion criteria included type 1 diabetes mellitus; a history of metabolic acidosis, including lactic acidosis, diabetic ketoacidosis, or diabetic coma; a history of myocardial infarction, unstable angina, coronary artery bypass graft surgery, stroke, or transient ischemic attack within 3 months before screening; New York Heart Association functional class III–IV heart failure; recent treatment with any type of insulin; use of medications known to affect glycemia, such as systemic steroids or immunosuppressants; use of weight-reducing medications; and an estimated glomerular filtration rate (eGFR) <30 mL/min/1.73 m². Patients with recent antibiotic use or other conditions that could significantly alter gut microbiota composition were excluded where applicable.

### Study design and procedure

This was a 12-week, single-center, randomized, open-label, parallel-group, active-controlled pilot study with a 24-week observational extension phase, designed to evaluate preliminary efficacy and generate hypotheses for future larger-scale studies (Clinical Research Information Service, KCT0004923). The study was conducted at Kyung Hee University Hospital at Gangdong, Republic of Korea, between October 2019 and December 2022. Randomization was performed centrally using an interactive web response system with a block size of four. Eligible patients were randomly assigned in a 1:1 ratio to receive either dulaglutide 0.75 mg once weekly (subcutaneously) or empagliflozin 10 mg once daily (orally) as add-on therapy (**Supplementary Figure 1A**).

All participants received standardized counseling on lifestyle modification, including dietary habits and physical activity for weight management, at visit 1. At week 4, all participants were successfully uptitrated to dulaglutide 1.5 mg/week (13/13, 100%) and empagliflozin 25 mg/day (12/12, 100%), with no dose reductions due to tolerability issues, and these doses were maintained until week 12 (**Supplementary Figure 1A**). Participants with medication adherence below 80% were not included in the per-protocol analysis (16). The primary efficacy analysis was performed in the per-protocol population, including participants who completed the 12-week treatment period without major protocol deviations. Treatment adherence and protocol compliance were monitored throughout the study period. In cases of symptomatic or potential hypoglycemia, the dose of glimepiride was reduced at week 4 at the investigator’s discretion according to standard clinical practice. After completion of the 12-week treatment period, participants were followed until week 36 as part of the observational extension phase, provided there were no changes in their antidiabetic medication regimen (**Supplementary Figure 1A**). Data obtained during the extension phase were analyzed in an exploratory manner using available data only.

### Efficacy assessments

The primary efficacy endpoint was the change in HbA1c from baseline at week 12. Secondary efficacy endpoints included the proportion of patients achieving HbA1c <7.0%, and changes from baseline at week 12 in fasting plasma glucose (FPG), glycated albumin (GA), fasting insulin, homeostasis model assessment of insulin resistance (HOMA-IR), and β-cell function (HOMA-β). HOMA-IR and HOMA-β were calculated as follows (17): HOMA-IR = FPG (mg/dL) × fasting insulin (μU/mL)/405; HOMA-β = 360 × fasting insulin (μU/mL)/[FPG (mg/dL) − 63]. Additional secondary endpoints included changes from baseline at week 12 in body weight, body mass index (BMI), waist circumference (WC), body fat percentage, muscle mass, subcutaneous fat area, and visceral fat area, as well as the proportion of patients achieving ≥3.0% weight loss. Body composition was assessed using bioelectrical impedance analysis (InBody720; InBody Co., Seoul, Korea). Abdominal computed tomography (CT) was used to quantify visceral and subcutaneous adipose tissue areas (18).

### Exploratory metabolic and gut microbiome analyses

Exploratory endpoints included changes in HbA1c, FPG, body weight, and BMI at weeks 24 and 36 among participants who continued the assigned treatment during the post-trial observational period. Additional exploratory analyses evaluated treatment-related changes in bile acid composition, plasma metabolites, and gut microbiota after 12 weeks of therapy.

Serum bile acids were extracted and quantified using liquid chromatography–tandem mass spectrometry (LC–MS/MS). Briefly, serum samples (50 μL) were mixed with internal standards and processed according to a validated protocol. SCFAs and BCAAs were analyzed following derivatization and LC–MS/MS analysis, with serum samples (100 μL) mixed with acetonitrile containing isotopically labeled internal standards.

Gut microbiota composition was analyzed using 16S rRNA gene sequencing. Microbial DNA was extracted from fecal samples collected at baseline and week 12. Sequencing of the V3–V4 hypervariable regions was performed, followed by standard quality-control filtering procedures to remove low-quality reads and potential sequencing artifacts. Taxonomic assignment was conducted using the SILVA reference database. Low-abundance taxa detected in only a small proportion of samples were excluded prior to downstream analysis. Alpha diversity indices were calculated to assess microbial richness and evenness. Beta diversity was evaluated based on Bray–Curtis dissimilarity and visualized using non-metric multidimensional scaling (NMDS). Differentially abundant taxa were identified using linear discriminant analysis effect size (LEfSe) with a LDA score threshold of 2.0 and P < 0.05. Correlation analyses between microbial taxa and metabolic parameters were exploratory and hypothesis-generating in nature.

### Safety assessments

Safety endpoints included treatment-emergent adverse events (TEAEs), adverse drug reactions (ADRs), hypoglycemic episodes, urinary tract infections, genital infections, polyuria, vital signs, clinical laboratory parameters, and electrocardiographic findings during the treatment period.

### Statistical analysis

Baseline characteristics of the study participants were summarized as means with standard deviations, medians with interquartile ranges, or numbers with percentages, as appropriate. Between-group comparisons of continuous variables were performed using Student’s *t* test or the Mann–Whitney *U* test, while categorical variables were compared using the χ² test. A formal sample size calculation was not performed, as this study was designed as a pilot trial to explore preliminary efficacy and estimate effect sizes for future studies. Accordingly, effect sizes, directionality, and clinical relevance were considered when interpreting the results.

The primary efficacy endpoint was analyzed using a mixed-effects model for repeated measures (MMRM), with change in HbA1c at week 12 as the dependent variable. The MMRM included treatment group, visit, and the interaction between treatment group and visit as fixed effects. Secondary efficacy and exploratory endpoints were analyzed using MMRM, generalized linear mixed models for categorical outcomes, or analysis of covariance (ANCOVA), as appropriate. Least-squares (LS) means with standard errors (SEs), 95% confidence intervals (CIs), and P values were calculated for each treatment group and for between-group comparisons. No adjustment for multiple comparisons was performed for secondary and exploratory endpoints.

Exploratory analyses of bile acid profiles and plasma metabolites were conducted by comparing changes from baseline to week 12 between treatment groups using nonparametric tests, given the exploratory nature of these outcomes. Gut microbiome data were analyzed using standard bioinformatic and statistical pipelines. Alpha diversity indices were compared between groups using the Wilcoxon rank-sum test. Beta diversity was assessed based on Bray–Curtis dissimilarity and visualized using non-metric multidimensional scaling (NMDS), with between-group differences evaluated by permutational multivariate analysis of variance (PERMANOVA). Differentially abundant taxa were identified using linear discriminant analysis effect size (LEfSe).

Safety analyses were performed by comparing the incidence of adverse events between treatment groups. Because of the exploratory pilot nature of the study and the limited sample size, missing values were not imputed, and analyses were performed using available data. Because only one participant was lost to follow-up before week 12, the primary analysis was conducted in participants who completed the treatment period. All statistical analyses were conducted using R software (version 3.2.1).

## Results

### Baseline characteristics

A total of 26 patients were screened, and 25 were randomized to receive dulaglutide 0.75 mg subcutaneously (n = 13) or empagliflozin 10 mg once daily orally (n = 12). All patients in the dulaglutide group and 11 patients in the empagliflozin group completed the 12-week treatment period (**Supplementary Figure 1B**). One participant in the empagliflozin group was lost to follow-up before week 12 and was excluded from the efficacy analysis. Among these, 12 and 10 patients, respectively, were followed until week 36. One patient in each group discontinued the assigned treatment because of a change in antidiabetic medication.

Baseline characteristics were largely comparable between groups (**Table 1**), except for systolic blood pressure [SBP] (124.9 ± 9.8 vs. 134.0 ± 9.6 mmHg, P = 0.033) and aspartate aminotransferase [AST] levels (27.5 ± 8.0 vs. 45.8 ± 23.6 U/L, P = 0.031). The mean age of the study population was 56.6 ± 8.0 years, 50.0% were male, the mean BMI was 27.4 ± 3.6 kg/m², the mean diabetes duration was 7.9 ± 4.5 years, and the mean baseline HbA1c level was 9.5 ± 1.0%.

**Table 1:** Baseline clinical and metabolic characteristics of study participants.

Variables	Total (N = 24)	Dulaglutide (N = 13)	Empagliflozin (N = 11)	P value
Age (years)	56.6 ± 8.0	56.3 ± 7.7	56.9 ± 8.7	0.858
Men, n (%)	12 (50.0)	5 (38.5)	7 (63.6)	0.413
Duration of diabetes (year)	7.9 ± 4.5	8.2 ± 5.2	7.6 ± 3.8	0.768
SBP (mmHg)	129.1 ± 10.6	124.9 ± 9.8	134.0 ± 9.6	0.033
DBP (mmHg)	77.5 ± 9.0	75.8 ± 10.4	79.4 ± 7.0	0.353
Body weight, total (kg)	73.4 ± 12.7	71.2 ± 13.5	76.0 ± 11.8	0.371
Body weight, male (kg)	79.8 ± 10.1	74.4 ± 13.1	83.6 ± 5.8	0.122
Body weight, female (kg)	67.0 ± 12.1	69.2 ± 14.3	62.6 ± 4.2	0.391
BMI (kg/m^2^)	27.4 ± 3.6	27.2 ± 4.3	27.6 ± 2.8	0.796
WC, total (cm)	96.1 ± 8.6	95.1 ± 9.6	97.4 ± 7.6	0.532
WC, male (cm)	98.7 ± 6.6	95.0 ± 6.7	101.4 ± 5.5	0.098
WC, female (cm)	93.5 ± 9.8	95.2 ± 11.4	90.3 ± 5.3	0.442
Body fat ratio (%)	33.1 ± 6.2	32.6 ± 6.9	33.6 ± 5.4	0.727
Abdomen fat ratio (%)	0.9 ± 0.1	0.9 ± 0.1	1.0 ± 0.1	0.155
Muscle mass (kg)	27.0 ± 5.4	26.2 ± 5.3	28.0 ± 5.7	0.446
Subcutaneous fat (cm^3^)	175.9 ± 51.1	170.6 ± 61.2	182.3 ± 37.7	0.574
Visceral fat (cm^3^)	165.4 ± 50.5	148.0 ± 43.0	185.9 ± 52.9	0.063
Visceral-subcutaneous fat ratio	1.0 ± 0.4	1.0 ± 0.5	1.0 ± 0.3	0.424
FPG (mg/dL)	202.3 ± 45.9	191.8 ± 44.5	214.7 ± 46.5	0.232
HbA1c (%)	9.5 ± 1.0	9.6 ± 0.9	9.4 ± 1.3	0.651
HbA1c >9.0%, n (%)	18 (75.0)	10 (76.9)	8 (72.7)	0.813
Glycated albumin (%)	25.0 ± 4.7	25.0 ± 4.9	25.1 ± 4.5	0.962
eGFR (mL/min/1.73 m^2^)	94.0 ± 20.2	95.9 ± 23.0	91.7 ± 16.9	0.622
AST (U/L)	35.9 ± 19.0	27.5 ± 8.0	45.8 ± 23.6	0.031
ALT (U/L)	43.7 ± 23.4	36.4 ± 13.3	52.3 ± 29.9	0.127
HS-CRP (mg/dL)	1.6 ± 1.8	1.6 ± 1.9	1.6 ± 1.7	0.252
Total cholesterol (mg/dL)	149.4 ± 28.2	149.8 ± 32.5	148.9 ± 23.6	0.937
TG (mg/dL)	196.0 ± 116.7	197.9 ± 137.1	193.7 ± 93.8	0.685
HDL-C (mg/dL)	46.5 ± 11.4	46.8 ± 13.0	46.0 ± 10.0	0.861
LDL-C (mg/dL)	80.8 ± 20.9	78.4 ± 22.6	83.7 ± 19.4	0.545
Insulin (uIU/mL)	8.8 ± 4.4	9.5 ± 5.1	8.1 ± 3.6	0.441
C-peptide (ng/mL)	2.4 ± 0.7	2.4 ± 0.7	2.3 ± 0.8	0.765
HOMA-IR	4.5 ± 2.8	4.7 ± 3.2	4.3 ± 2.3	1.000
HOMA-β (%)	25.6 ± 15.7	29.9 ± 18.6	20.5 ± 9.9	0.132
Hypertension, n (%)	11 (45.8)	6 (46.2)	5 (45.5)	1.000
Dyslipidemia, n (%)	22 (91.7)	12 (92.3)	10 (90.9)	0.902
Metformin dosage, mg/day	1,250.0 ± 373.0	1,369.2 ± 427.0	1,109.1 ± 246.8	0.102
Glimepiride dosage, mg/day	6.0 ± 1.8	6.2 ± 1.9	5.9 ± 1.8	0.804

SBP, systolic blood pressure; DBP, diastolic blood pressure; BMI, body mass index; FPG, fasting plasma glucose; eGFR, estimated glomerular filtration rate; AST, aspartate aminotransferase; ALT, alanine transaminase; HS-CRP, high sensitivity C-reactive protein; TG, triglycerides; HDL-C, high-density lipoprotein cholesterol; LDL-C, low-density lipoprotein cholesterol; HOMA-IR, homeostatic model assessment of insulin resistance; HOMA-β, homeostatic model assessment of beta cell function.

### Primary efficacy endpoint

**Figure 1:**
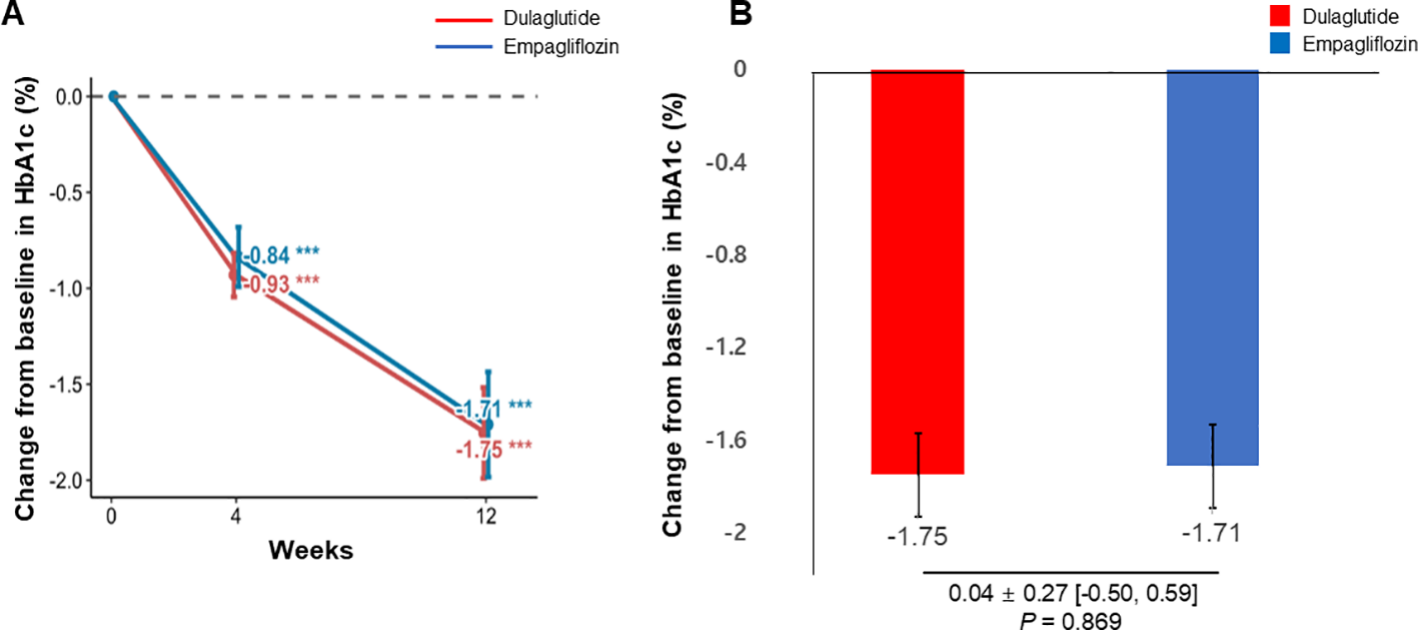
Changes in HbA1c after 12 weeks of treatment. **(A)** Mean change in HbA1c over time at weeks 4 and 12. **(B)** Least squares mean change in HbA1c at week 12. *P < 0.05, **P < 0.01, and ***P < 0.001vs. baseline within group.

At week 12, HbA1c levels were significantly reduced from baseline in both the dulaglutide group (−1.75 ± 0.18%, P < 0.001) and the empagliflozin group (−1.71 ± 0.20%, P < 0.001; **Figure 1A** and **Table 2**). No statistically significant between-group difference in HbA1c reduction was observed (LS mean difference, 0.04%; 95% CI, −0.50 to 0.59; P = 0.869; **Figure 1B**; **Table 2**).

**Table 2:** Changes in glycemic parameters after 12 weeks of treatment.

Parameters	Dulaglutide (N = 13)	Empagliflozin (N = 11)
HbA1c (%)		
Change from baseline, LS mean ± SE	-1.75 ± 0.18	-1.71 ± 0.20
LS mean difference [95% CI]	0.04 ± 0.27 [-0.50, 0.59]	
P for comparing two groups	0.869	
P for comparing baseline and week 12	<0.001	<0.001
Fasting plasma glucose (mg/dL)		
Change from baseline, LS mean ± SE	-42.15 ± 10.00	-69.09 ± 10.90
LS mean difference [95% CI]	-26.94 ± 14.80 [-56.80, 2.93]	
P for comparing two groups	0.076	
P for comparing baseline and week 12	<0.001	<0.001
Glycated albumin (%)		
Change from baseline, LS mean ± SE	-6.15 ± 0.74	-7.77 ± 0.81
LS mean difference [95% CI]	-1.63 ± 1.09 [-3.83, 0.58]	
P for comparing two groups	0.144	
P for comparing baseline and week 12	<0.001	<0.001
Insulin (uIU/mL)		
Change from baseline, LS mean ± SE	2.22 ± 1.07	-0.79 ± 1.16
LS mean difference [95% CI]	-3.01 ± 1.57 [-6.19, 0.16]	
P for comparing two groups	0.062	
P for comparing baseline and week 12	0.043	0.498
HOMA-IR		
Change from baseline, LS mean ± SE	-0.19 ± 0.59	-1.71 ± 0.64
LS mean difference [95% CI]	-1.53 ± 0.87 [-3.28, 0.23]	
P for comparing two groups	0.086	
P for comparing baseline and week 12	0.754	0.011
HOMA-β (%)		
Change from baseline, LS mean ± SE	25.42 ± 11.40	23.17 ± 12.40
LS mean difference [95% CI]	-2.26 ± 16.80 [-36.20, 31.70]	
P for comparing two groups	0.894	
P for comparing baseline and week 12	0.031	0.068

LS; least squares; SE, standard error; HOMA-IR, homeostasis model assessment of insulin resistance.

### Secondary efficacy endpoints

**Figure 2:**
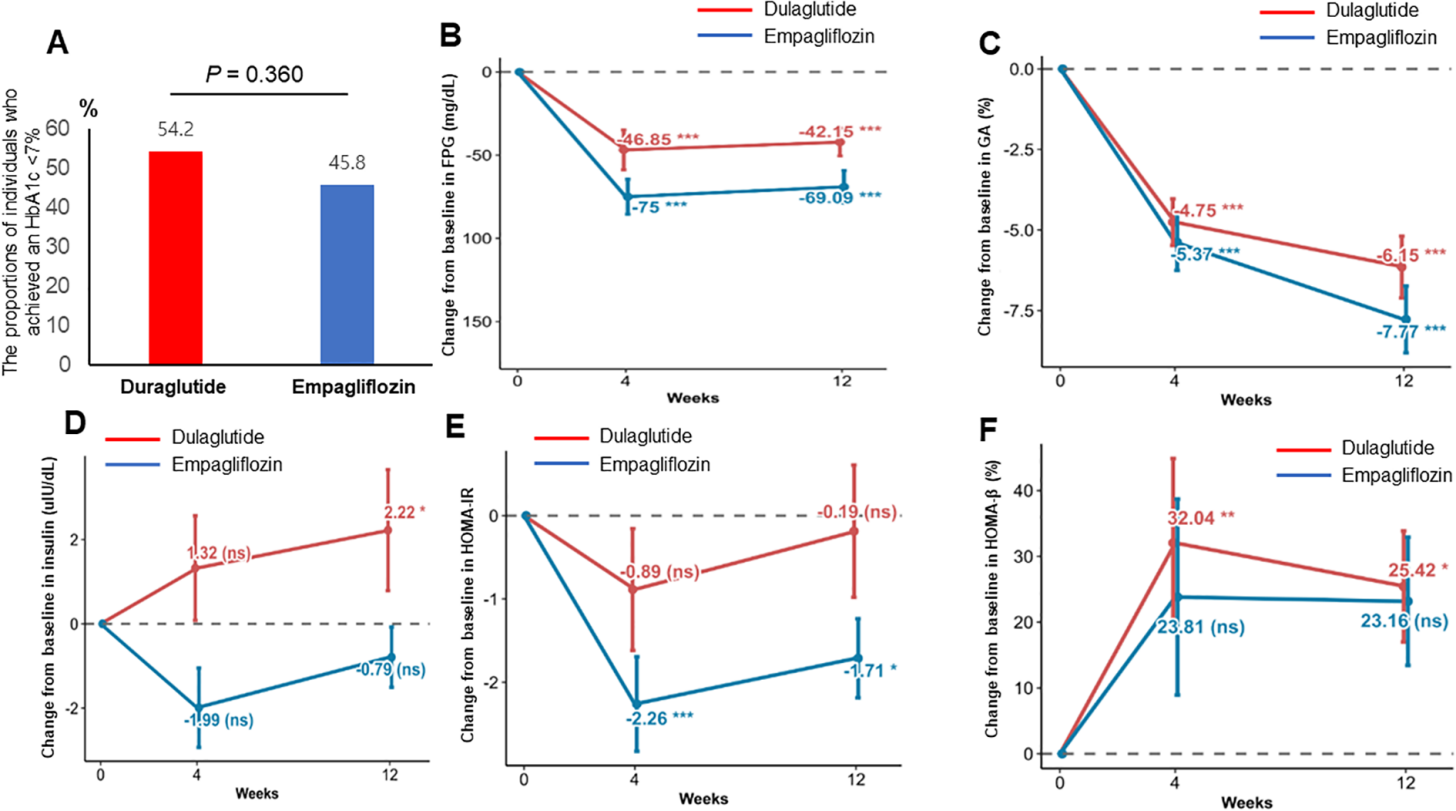
Changes in glycemic and insulin-related parameters after 12 weeks of treatment. **(A)** Proportion of participants achieving HbA1c <7.0% at week 12. **(B)** Change in fasting plasma glucose (%). **(C)** Change in glycated albumin (%). **(D)** Change in fasting insulin levels (μIU/mL). **(E)** Change in homeostasis model assessment of insulin resistance. **(F)** Change in homeostasis model assessment of β-cell function. Data are presented as least squares means ± standard error. ^*^P < 0.05, ^**^P < 0.01, and ^***^P < 0.001 vs. baseline within group.

The proportion of participants achieving HbA1c <7.0% at week 12 did not differ between the dulaglutide and empagliflozin groups (54.2% vs. 45.8%, P = 0.360; **Figure 2A**). Fasting plasma glucose and glycated albumin levels were significantly reduced from baseline in both groups, with no significant between-group differences (**Table 2**; **Figures 2B, C**). Plasma insulin levels increased after 12 weeks of dulaglutide treatment, whereas no significant change was observed with empagliflozin, accompanied by an increase in HOMA-β in the dulaglutide group and a decrease in HOMA-IR in the empagliflozin group (**Table 2**; **Figures 2D–F**); however, none of these parameters differed significantly between groups.

**Figure 3:**
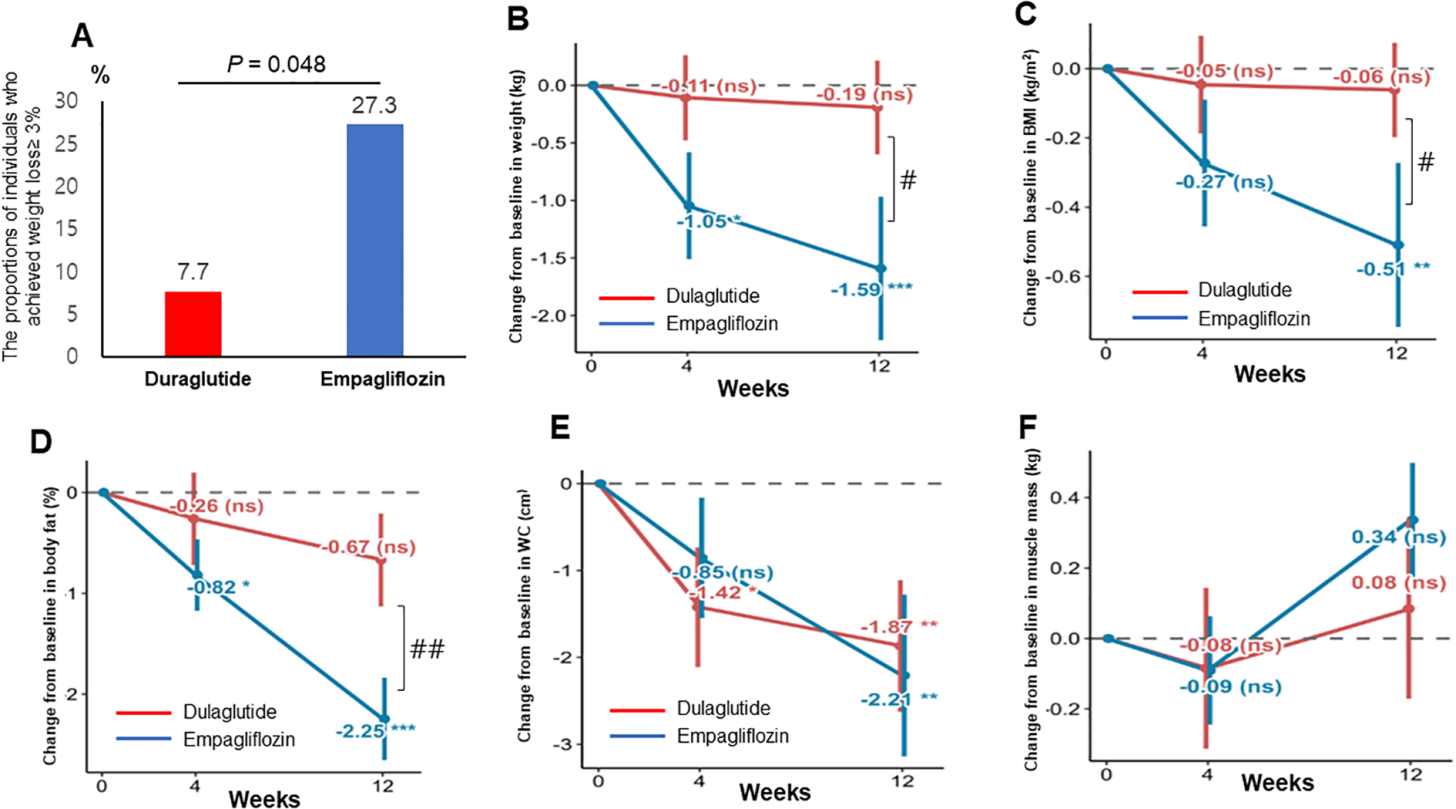
Changes in body weight and body composition after 12 weeks of treatment **(A)** Proportion of participants achieving ≥3.0% body weight reduction at week 12. **(B)** Change in body weight (kg). **(C)** Change in body mass index (kg/m^2^). **(D)** Change in body fat mass (kg). **(E)** Change in waist circumference (cm). **(F)** Change in total muscle mass (kg). Data are presented as least squares means ± standard error. ^*^P < 0.05, ^**^P < 0.01, and ^***^P < 0.001 vs. baseline within group; ^#^ and ^##^ indicate between-group differences (dulaglutide vs. empagliflozin, P < 0.05 and P < 0.01, respectively).

At week 12, a greater proportion of participants in the empagliflozin group achieved ≥3.0% weight loss compared with the dulaglutide group (27.3% vs. 7.7%, P = 0.048; **Figure 3A**). Empagliflozin was associated with significantly greater reductions in body weight, BMI, and body fat than dulaglutide (**Table 3**; **Figures 3B–D**), whereas WC decreased similarly in both groups (**Figure 3E**). No significant changes were observed in total muscle mass (**Figure 3F**), subcutaneous fat area, or visceral fat/subcutaneous fat ratio in either group, although visceral fat area showed a nonsignificant trend toward reduction in the empagliflozin group (P = 0.087). While HbA1c decreased in nearly all participants in both groups, a higher proportion of patients in the empagliflozin group experienced body weight reduction at week 12 (**Supplementary Figure 2**).

**Table 3:** Changes in body weight and body composition after 12 weeks of treatment.

Parameters	Dulaglutide (N = 13)	Empagliflozin (N = 11)
Body weight (kg)		
Change from baseline, LS mean ± SE	-0.19 ± 0.39	-1.59 ± 0.42
LS mean difference [95% CI]	-1.40 ± 0.57 [-2.55, -0.25]	
P for comparing two groups	0.018	
P for comparing baseline and week 12	0.621	<0.001
BMI (kg/m^2^)		
Change from baseline, LS mean ± SE	-0.06 ± 0.15	-0.51 ± 0.16
LS mean difference [95% CI]	-0.45 ± 0.21 [-0.88, -0.02]	
P for comparing two groups	0.042	
P for comparing baseline and week 12	0.673	0.002
Body fat (%)		
Change from baseline, LS mean ± SE	-0.67 ± 0.37	-2.25 ± 0.40
LS mean difference [95% CI]	-1.58 ± 0.54 [-2.67, -0.49]	
P for comparing two groups	0.006	
P for comparing baseline and week 12	0.075	<0.001
WC (cm)		
Change from baseline, LS mean ± SE	-1.87 ± 0.62	-2.21 ± 0.68
LS mean difference [95% CI]	-0.34 ± 0.92 [-2.20, 1.52]	
P for comparing two groups	0.714	
P for comparing baseline and week 12	0.005	0.002
Total muscle mass (kg)		
Change from baseline, LS mean ± SE	0.08 ± 0.18	0.34 ± 0.19
LS mean difference [95% CI]	0.25 ± 0.26 [-0.27, 0.78]	
P for comparing two groups	0.339	
P for comparing baseline and week 12	0.634	0.086

LS; least squares; SE, standard error; BMI, body mass index; WC, waist circumference.

### Exploratory endpoints – changes in extension period

During the observational extension period, reductions in HbA1c were sustained through week 36 in both treatment groups (**Supplementary Figure 3A**). The LS mean ± SE change in HbA1c from baseline at week 36 did not differ between the dulaglutide and empagliflozin groups (−1.48% vs. −1.84%, P = 0.307; **Supplementary Table 1**). FPG levels did not differ between groups through week 12 but were lower in the empagliflozin group at week 24 and remained significantly lower at week 36 (**Supplementary Figure 3B**; **Supplementary Table 1**). Body weight and BMI decreased after week 24 in the dulaglutide group and were maintained through week 36, whereas reductions achieved with empagliflozin were sustained throughout the extension period (**Supplementary Figures 3C, D**). However, no significant between-group differences in body weight or BMI were observed at weeks 24 or 36 (**Supplementary Table 1**).

### Exploratory endpoints – changes in plasma metabolites

Exploratory analysis of plasma bile acids demonstrated a selective change in primary bile acid composition following dulaglutide treatment (**Supplementary Figure 4**). Cholic acid levels increased significantly at week 12 compared with baseline in the dulaglutide group, whereas no significant changes were observed in other bile acid species, including deoxycholic acid, glycocholic acid, glycochenodeoxycholic acid, or lithocholic acid (**Supplementary Figures 4A–F**). In contrast, empagliflozin treatment was not associated with significant changes in circulating bile acid levels over the 12-week treatment period.

Analysis of gut-derived fatty acids revealed divergent patterns between treatment groups (**Supplementary Figure 5**). Plasma concentrations of short-chain fatty acids, including acetic, propionic, and butyric acids, decreased at week 12 in the dulaglutide group, whereas these metabolites tended to increase following empagliflozin treatment (**Supplementary Figures 5A–D**). A similar pattern was observed for branched-chain fatty acids, including isobutyric, 2-methylbutyric, isovaleric, and valeric acids, which decreased after dulaglutide treatment but showed overall increases after empagliflozin treatment (**Supplementary Figures 5E–H**).

Among branched-chain amino acids, no significant changes were observed in leucine or isoleucine following 12 weeks of treatment in either group (**Supplementary Figures 6A–D**). Valine levels increased modestly in both treatment groups at week 12, with a greater relative increase observed in the empagliflozin group compared with the dulaglutide group (**Supplementary Figure 6B**). Levels of 2-aminobutyric acid remained unchanged in both groups (**Supplementary Figure 6E**).

### Exploratory endpoints – changes in gut microbiome

Exploratory analysis of gut microbiota revealed no significant changes in overall alpha diversity indices, including richness and evenness, after 12 weeks of treatment in either the dulaglutide or empagliflozin group (**Supplementary Figure 7A**). Similarly, beta diversity analysis based on Bray–Curtis dissimilarity showed substantial overlap in microbial community composition between baseline and week 12 in both treatment groups, with no distinct clustering observed (**Supplementary Figure 7B**). Despite the absence of community-wide alterations, differential abundance analysis identified several taxa exhibiting treatment-associated changes. In the dulaglutide group, multiple bacterial taxa were differentially enriched at week 12 compared with baseline, whereas in the empagliflozin group, a more limited number of taxa showed differential abundance (**Supplementary Table 2**).

**Figure 4:**
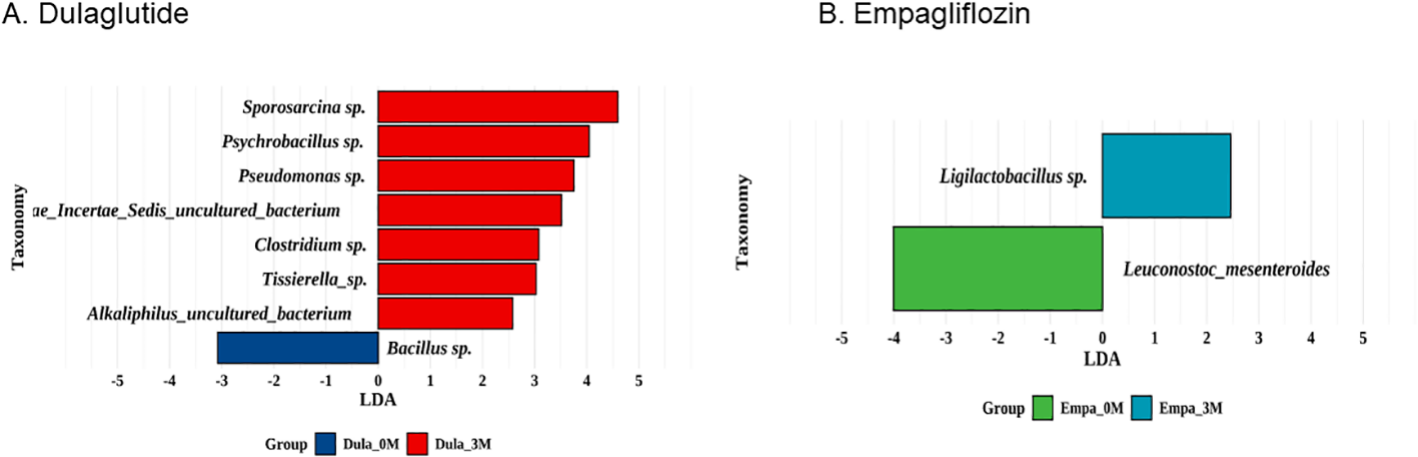
Differentially abundant gut microbial taxa identified by LEfSe analysis. **(A)** Taxa showing significant differential abundance between baseline and post-treatment time points in the dulaglutide group. **(B)** Taxa showing significant differential abundance between baseline and post-treatment time points in the empagliflozin group. Bars represent linear discriminant analysis (LDA) scores, indicating the effect size of each taxon. Positive and negative LDA scores reflect enrichment at post-treatment or baseline, respectively. Only taxa with an LDA score >2.0 and P < 0.05 are shown.

**Figure 5:**
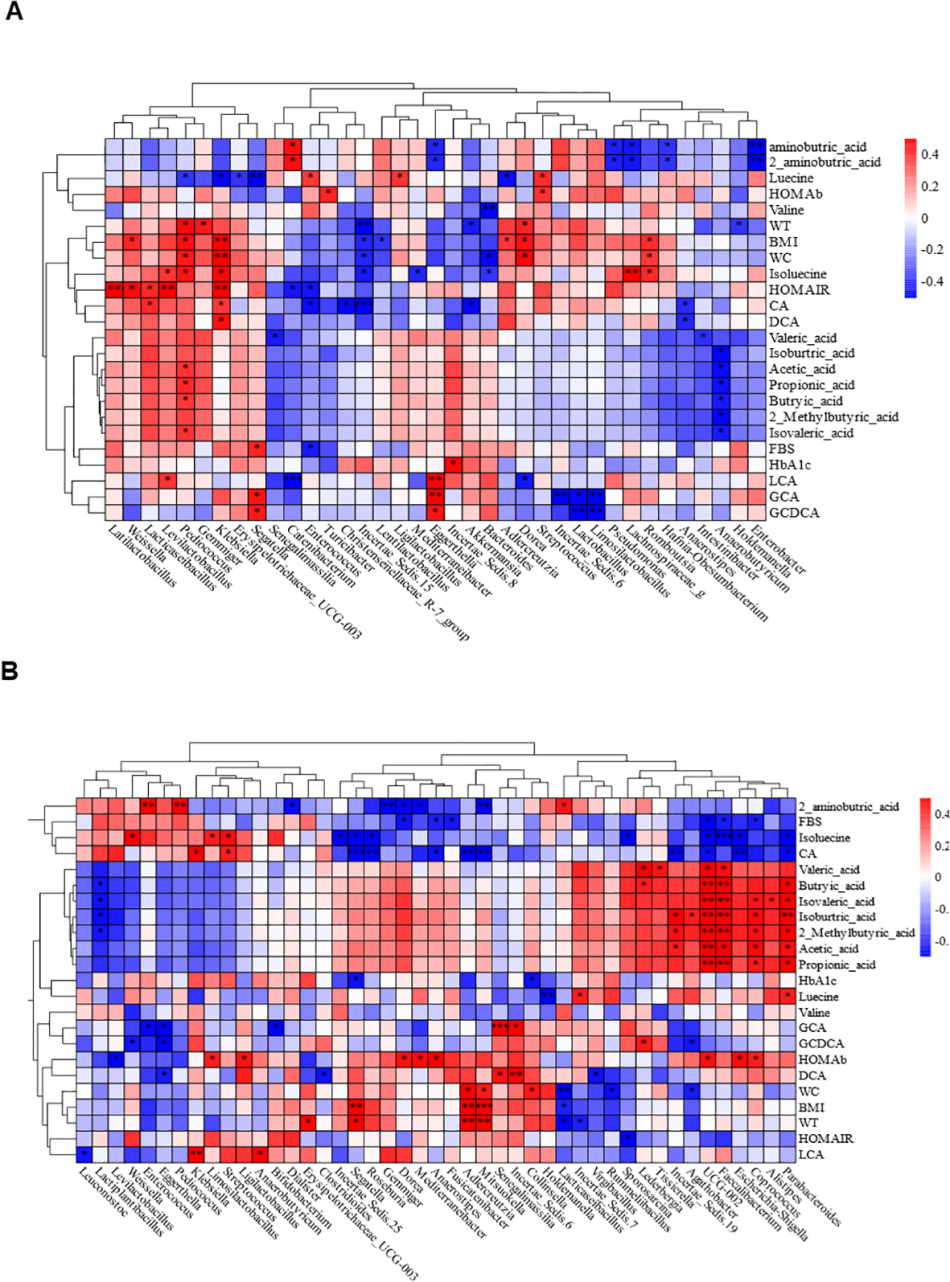
Correlation analysis between gut microbiota and metabolic parameters. **(A)** Correlation matrix in the dulaglutide-treated group. **(B)** Correlation matrix in the empagliflozin-treated group. Heatmaps display Spearman correlation coefficients between relative abundances of selected gut microbial taxa and metabolic variables, including glycemic indices, body composition parameters, bile acids, short-chain and branched-chain fatty acids, and branched-chain amino acids. Red and blue colors indicate positive and negative correlations, respectively, with color intensity reflecting correlation strength. Hierarchical clustering was applied to both microbial taxa and metabolic parameters. Only statistically significant correlations (P < 0.05) are indicated. Correlation analyses were exploratory in nature. *P < 0.05, **P < 0.01, and ***P < 0.001.

Differential abundance analysis identified treatment-specific taxonomic changes after 12 weeks. LEfSe-based LDA analysis identified enrichment of *Sporosarcina* sp. and *Psychrobacillus* sp. with depletion of *Bacillus* sp. in the dulaglutide group (**Figure 4A**), whereas the empagliflozin group showed minimal overall changes, with increased *Leuconostoc mesenteroides* and decreased *Ligilactobacillus* sp. after treatment (**Figure 4B**). Correlation analysis further demonstrated distinct association patterns between gut microbiota and host metabolic parameters according to treatment. In the dulaglutide group (**Figure 5A**), microbial taxa were primarily associated with insulin-related indices (e.g., HOMA-IR and HOMA-β) and bile acid components, whereas in the empagliflozin group (**Figure 5B**), taxa were more strongly associated with body weight–related parameters (e.g., body weight, BMI, and WC) and short-chain and branched-chain fatty acids.

### Safety outcomes

The incidence of TEAEs was comparable between the dulaglutide and empagliflozin groups (**Supplementary Table 2**). TEAEs were reported in 23.1% (n = 3) of participants receiving dulaglutide and 25.0% (n = 3) of those receiving empagliflozin, and most events were mild in severity. No treatment-emergent serious adverse events or adverse events leading to study discontinuation were observed. Symptomatic hypoglycemic episodes were infrequent and mild, occurring in one participant (7.7%) in the dulaglutide group and two participants (16.7%) in the empagliflozin group (**Supplementary Table 3**). Glimepiride dose reduction at week 4 was required in 2 participants (15.4%) in the dulaglutide group and 1 participant (9.1%) in the empagliflozin group because of symptomatic or potential hypoglycemia.

Changes in renal function were additionally evaluated. Estimated glomerular filtration rate (eGFR) remained stable in both treatment groups at week 12, with no significant between-group differences. During the extension period, eGFR showed a similar trend, although data at week 36 were available only in a subset of participants (**Supplementary Table 4**).

## Discussion

In this preliminary head-to-head study of patients with T2DM inadequately controlled with metformin and sulfonylurea, both dulaglutide and empagliflozin significantly reduced HbA1c, with no statistically significant between-group difference observed in this pilot study. Despite similar overall glycemic improvement, empagliflozin induced earlier reductions in body weight, BMI, and body fat without loss of muscle mass, whereas weight reduction with dulaglutide emerged more gradually and became comparable at later time points. Both treatments were well tolerated. Sulfonylurea dose reductions were slightly more frequent in the dulaglutide group but were few in number and were unlikely to have materially affected the overall efficacy or safety findings. Nevertheless, concomitant sulfonylurea therapy may represent a potential confounding factor and should be considered when interpreting the study findings. Exploratory analyses suggested potential, modest differences in bile acid composition, gut-derived metabolites, and microbiome–metabolic associations, which should be interpreted cautiously given the exploratory nature of the study.

The comparable glucose-lowering effects of dulaglutide and empagliflozin are consistent with prior studies, despite their fundamentally different mechanisms of action. GLP-1RAs improve glycemia primarily through enhancement of glucose-dependent insulin secretion, suppression of glucagon release, delayed gastric emptying, and appetite reduction (19), whereas SGLT2is lower plasma glucose by promoting urinary glucose excretion independently of insulin (20).

One of the most clinically relevant findings of this study is the differential trajectory of weight reduction between treatments. Empagliflozin induced earlier weight loss, whereas dulaglutide demonstrated a delayed but sustained effect, resulting in comparable weight outcomes at later time points. This temporal difference may have implications for individualized treatment selection in clinical practice and likely reflects the distinct physiological mechanisms of each drug class. Importantly, baseline SBP was higher in the empagliflozin group and decreased more prominently during follow-up, suggesting potential differences in baseline hemodynamic status, which may have influenced early weight changes. Given the osmotic diuretic effect of SGLT2is (21), early volume contraction may have partially contributed to the greater early reductions in body weight observed in this group, although this mechanism alone is unlikely to fully account for the observed differences in body composition.

Consistent with these mechanisms, empagliflozin improved insulin resistance (HOMA-IR), whereas dulaglutide enhanced β-cell function (HOMA-β), in line with their known pharmacological effects (19, 20). These differential effects may contribute to the distinct temporal patterns of weight and body fat reduction observed between treatments, with improvements in insulin sensitivity potentially facilitating earlier reductions in adiposity in the empagliflozin group, whereas enhancement of β-cell function may be associated with more gradual metabolic and body composition changes in the dulaglutide group. These findings may have clinical implications, suggesting that GLP-1RAs could be particularly beneficial in patients with impaired β-cell function, whereas SGLT2is may be more suitable for those with predominant insulin resistance and obesity. Furthermore, combination therapy targeting complementary mechanisms may represent a promising strategy for achieving more comprehensive metabolic control (22), which warrants further investigation.

In exploratory bile acid analyses, dulaglutide treatment was associated with a selective increase in circulating cholic acid, whereas no significant changes were observed in secondary or conjugated bile acids. In contrast, empagliflozin treatment was not associated with significant alterations in circulating bile acid profiles. The selective increase in cholic acid observed following dulaglutide treatment may reflect potential modulation of bile acid metabolism by GLP-1RAs. Previous studies suggest that GLP-1RAs may influence bile acid circulation through alterations in gallbladder motility, as well as through interactions with bile acid–related signaling pathways, including farnesoid X receptor (FXR)-mediated metabolic regulation (23, 24). In addition, potential involvement of enterohepatic circulation and bile acid–mediated signaling pathways, such as TGR5 activation (25), cannot be excluded. However, given the exploratory nature of these findings and the small sample size, the clinical relevance and underlying mechanisms remain uncertain. Moreover, baseline differences in AST were observed between groups; however, AST is not a liver-specific marker, and other hepatic parameters, including alanine aminotransferase, were not significantly different. Therefore, although the clinical significance of this imbalance remains uncertain, it should be considered when interpreting the observed changes in bile acid profiles.

Despite minimal changes in overall gut microbial diversity, exploratory analyses suggested potential, treatment-related differences in gut-derived metabolites and microbiome–metabolic associations. Dulaglutide treatment was associated with reductions in circulating short-chain and branched-chain fatty acids, whereas empagliflozin treatment showed a tendency toward increased levels of these metabolites. These observations may reflect differences in gastrointestinal physiology and nutrient availability (26–28); however, these interpretations remain speculative. Despite glycemic improvement, plasma valine levels increased modestly after treatment, suggesting that normalization of BCAA metabolism may lag behind improvements in blood glucose control (29, 30).

Although no significant changes were observed in overall alpha or beta diversity, differential abundance analysis identified modest, taxa-specific shifts following treatment, suggesting localized alterations in microbial composition rather than global restructuring of the gut microbiome. This apparent discrepancy may be explained by the fact that overall diversity metrics capture broad community structure, whereas differential abundance analysis can detect changes in specific taxa without altering global diversity indices. However, these findings should be interpreted with caution, as the limited sample size and exploratory design preclude definitive conclusions regarding treatment-specific microbial adaptations. The observed taxonomic changes may reflect transient or context-dependent responses rather than stable or clinically meaningful alterations. Correlation analyses revealed distinct, treatment-specific patterns of microbiome–metabolite associations. These patterns indicate differences in host–microbiome–metabolite interaction profiles between treatments, even in the absence of marked changes in overall microbial diversity (31–33). However, these findings are exploratory and do not imply causality.

From a clinical perspective, these findings suggest that both GLP-1RAs and SGLT2is are effective add-on therapies for patients with T2DM inadequately controlled with metformin and sulfonylurea. Empagliflozin may provide more rapid and early weight reduction, whereas dulaglutide may provide comparable metabolic benefits over a longer treatment duration (4, 34, 35). The exploratory metabolic and microbiome-related findings provide preliminary insights for individualized treatment strategies but require further validation in larger studies. Recent metabolomics research suggests that metabolic profiling may contribute to pathway characterization, biomarker discovery, and individualized therapeutic strategies in diabetes. However, methodological standardization and external validation remain necessary before translation into routine clinical practice (36).

This study has several limitations. The small sample size and short duration limit the generalizability of the findings. The study was not powered to detect definitive differences in secondary or exploratory endpoints. The exploratory metabolomic and microbiome analyses were hypothesis-generating and should be interpreted with caution. Dietary intake, energy expenditure, and other factors influencing gut microbiota were not systematically controlled and may have affected the results. In addition, albuminuria was not assessed in this study, limiting further evaluation of renal outcomes. Given the number of secondary and exploratory endpoints assessed without adjustment for multiple comparisons, there is an increased risk of type I error; therefore, some statistically significant findings may represent chance findings and should be interpreted with caution.

In conclusion, both dulaglutide and empagliflozin significantly improved glycemic control in patients with T2DM inadequately controlled with metformin and sulfonylurea therapy, with no significant between-group difference observed in this exploratory pilot study. While dulaglutide primarily enhanced insulin secretion, empagliflozin improved insulin sensitivity. Empagliflozin was associated with earlier and sustained weight loss, whereas weight reduction with dulaglutide emerged more gradually over time. Both treatments were well tolerated. Exploratory findings suggested potential metabolic differences beyond glucose lowering, which require validation in larger, adequately powered studies.

## Data Availability

The microbiome sequencing data generated during the current study have been deposited in the NCBI Sequence Read Archive (SRA) under BioProject accession number PRJNA1477682 (https://www.ncbi.nlm.nih.gov/bioproject/PRJNA1477682). Other datasets generated and analyzed during the current study are available from the corresponding author on reasonable request.

## Supplementary material

Supplementary Figure 1 Study design and participant disposition. **(A)** Schematic overview of the randomized, open-label study design. **(B)** Flow diagram showing patients’ disposition.

Supplementary Figure 2 Individual changes in glycemic control and body weight at week 12. Each point represents an individual participant treated with dulaglutide (red) or empagliflozin (blue). The x-axis indicates the absolute change in HbA1c (%), and the y-axis indicates the percentage change in body weight from baseline.

Supplementary Figure 3 Longitudinal changes in glycemic and anthropometric parameters during the observational extension period. **(A)** Change in HbA1c (%). **(B)** Change in FPG (mg/dL). **(C)** Change in body weight (kg). **(D)** Change in BMI (kg/m^2^). Data are presented as least squares means ± standard error. ^*^P <0.05, ^**^P <0.01, and ^***^P <0.001 for comparing baseline and follow-up value. ^#^P <0.05, ^##^P <0.01, and ^###^P <0.001 for the comparison of dulaglutide and empagliflozin.

Supplementary Figure 4 Changes in plasma bile acid composition after 12 weeks of treatment. **(A)** Summary of fold changes and statistical significance for individual plasma bile acids. **(B–F)** Box plots showing plasma concentrations of cholic acid **(B)**, deoxycholic acid **(C)**, glycocholic acid **(D)**, glycochenodeoxycholic acid **(E)**, and lithocholic acid **(F)** at baseline and week 12 in each treatment group. Data are presented as median with interquartile range. P values indicate within-group comparisons versus baseline.

Supplementary Figure 5 Changes in plasma short-chain (SCFA) and branched-chain fatty acids (BCFA) after 12 weeks of treatment. **(A)** Summary of fold changes and statistical significance for individual SCFAs and BCFAs. **(B–D)** Box plots showing plasma concentrations of acetic acid **(B)**, propionic acid **(C)**, and butyric acid **(D)**. **(E–H)** Box plots showing plasma concentrations of isobutyric acid **(E)**, 2-methylbutyric acid **(F)**, isovaleric acid **(G)**, and valeric acid **(H)** at baseline and week 12 in each treatment group. Data are presented as median with interquartile range. P values indicate within-group comparisons versus baseline.

Supplementary Figure 6 Changes in plasma branched-chain amino acids after 12 weeks of treatment. **(A)** Summary of fold changes and statistical significance for individual branched-chain amino acids. **(B–E)** Box plots showing plasma concentrations of valine **(B)**, isoleucine **(C)**, leucine **(D)**, and 2-aminobutyric acid **(E)** at baseline and week 12 in each treatment group. Data are presented as median with interquartile range. P values indicate within-group comparisons versus baseline.

Supplementary Figure 7 Gut microbiome diversity analysis. **(A)** Alpha diversity of the gut microbiota at baseline and after 12 weeks of treatment with dulaglutide or empagliflozin. Alpha diversity was assessed using the Chao1 index. **(B)** Beta diversity of the gut microbiota at baseline and after 12 weeks of treatment with dulaglutide or empagliflozin. Non-metric multidimensional scaling (NMDS) plots based on Bray–Curtis dissimilarity are shown for each treatment group.
