# Supplementary material for: Dulaglutide versus empagliflozin as add-on therapy to metformin and sulfonylurea in type 2 diabetes: a randomized pilot study with exploratory metabolomic and microbiome analyses
Source: Front Endocrinol (Lausanne). 2026 Jun 17;17:1843595. doi: 10.3389/fendo.2026.1843595 (PMC13318618; doi:10.3389/fendo.2026.1843595)
Supplement: Supplementary Figure 1 — Study design and participant disposition. (A) Schematic overview of the randomized, open-label study design. (B) Flow diagram showing patients’ disposition. [file Presentation1.pptx]

## Slide 1
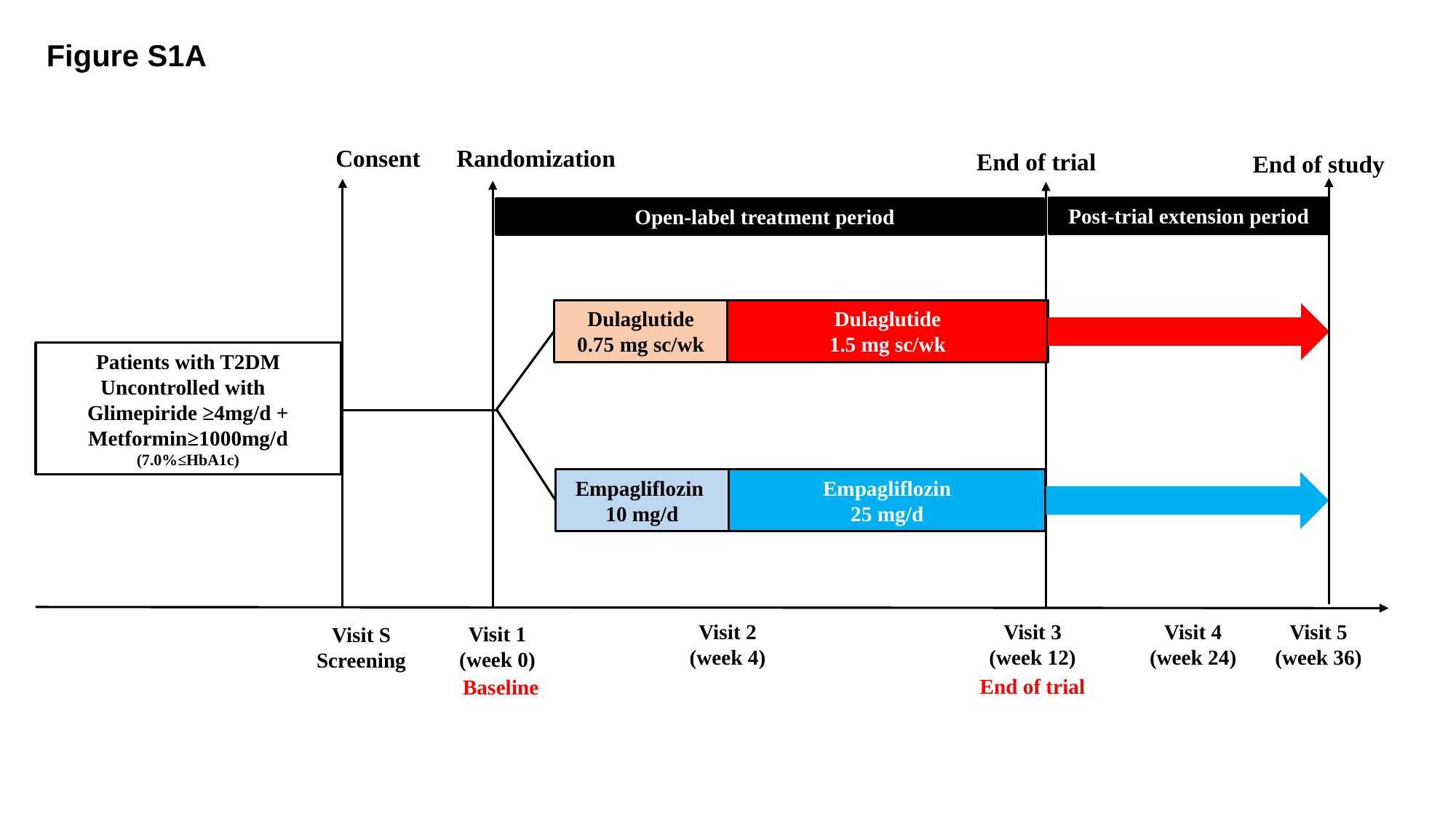

Figure S1A
Randomization
Consent
End of trial
End of study
Post-trial extension period
Open-label treatment period
Dulaglutide
1.5 mg sc/wk
Dulaglutide
0.75 mg sc/wk
Patients with T2DM
Uncontrolled with
Glimepiride ≥4mg/d + Metformin≥1000mg/d
(7.0%≤HbA1c)
Empagliflozin
25 mg/d
Empagliflozin
10 mg/d
Visit 2
(week 4)
Visit 4
(week 24)
Visit 5
(week 36)
Visit 3
(week 12)
Visit 1
(week 0)
Visit S
Screening
End of trial
Baseline

## Slide 2
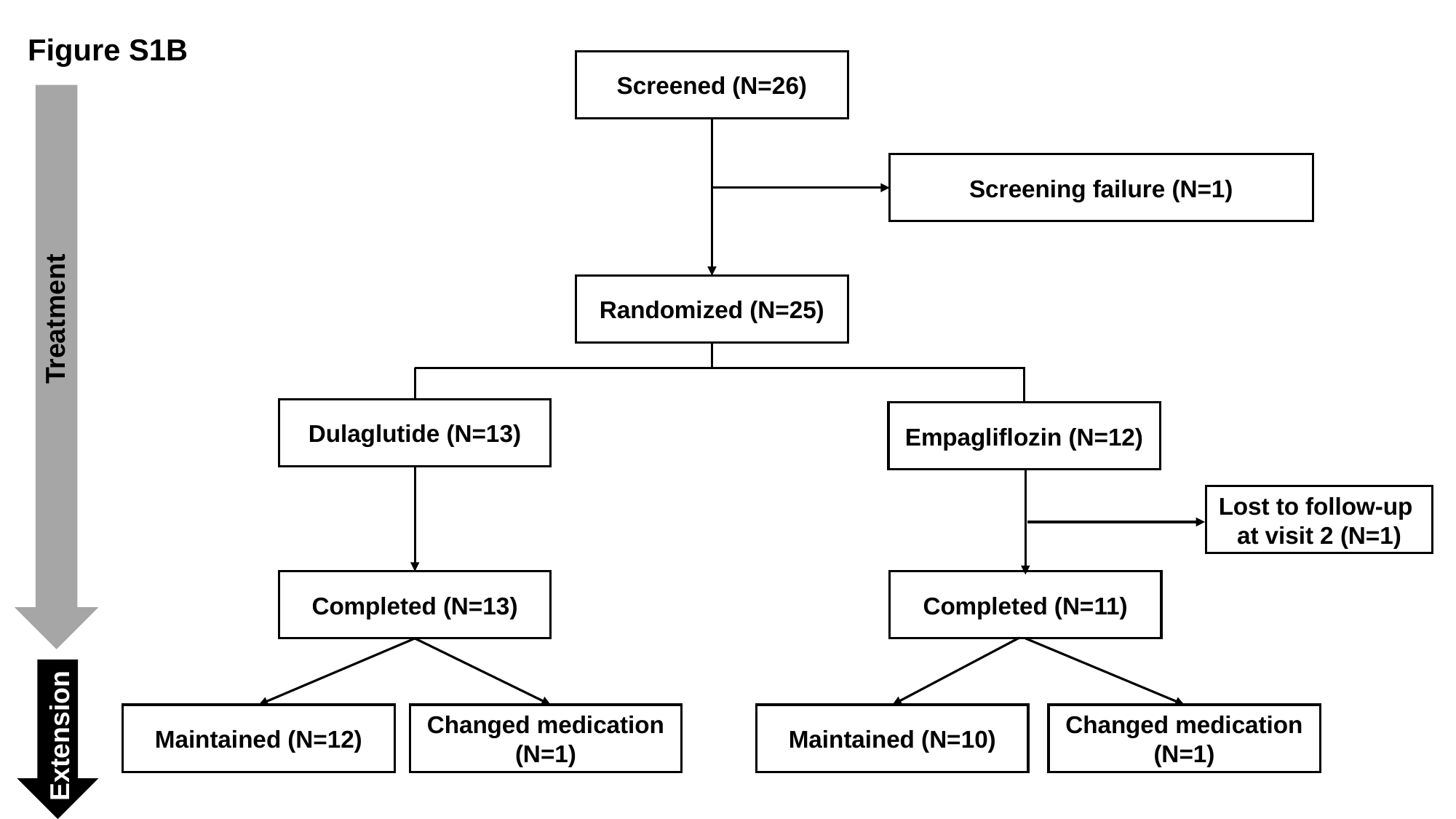

Figure S1B
Screened (N=26)
Screening failure (N=1)
Randomized (N=25)
Dulaglutide (N=13)
Empagliflozin (N=12)
Lost to follow-up
at visit 2 (N=1)
Completed (N=13)
Completed (N=11)
Maintained (N=10)
Maintained (N=12)
Changed medication (N=1)
Changed medication (N=1)
Treatment
Extension

## Slide 3
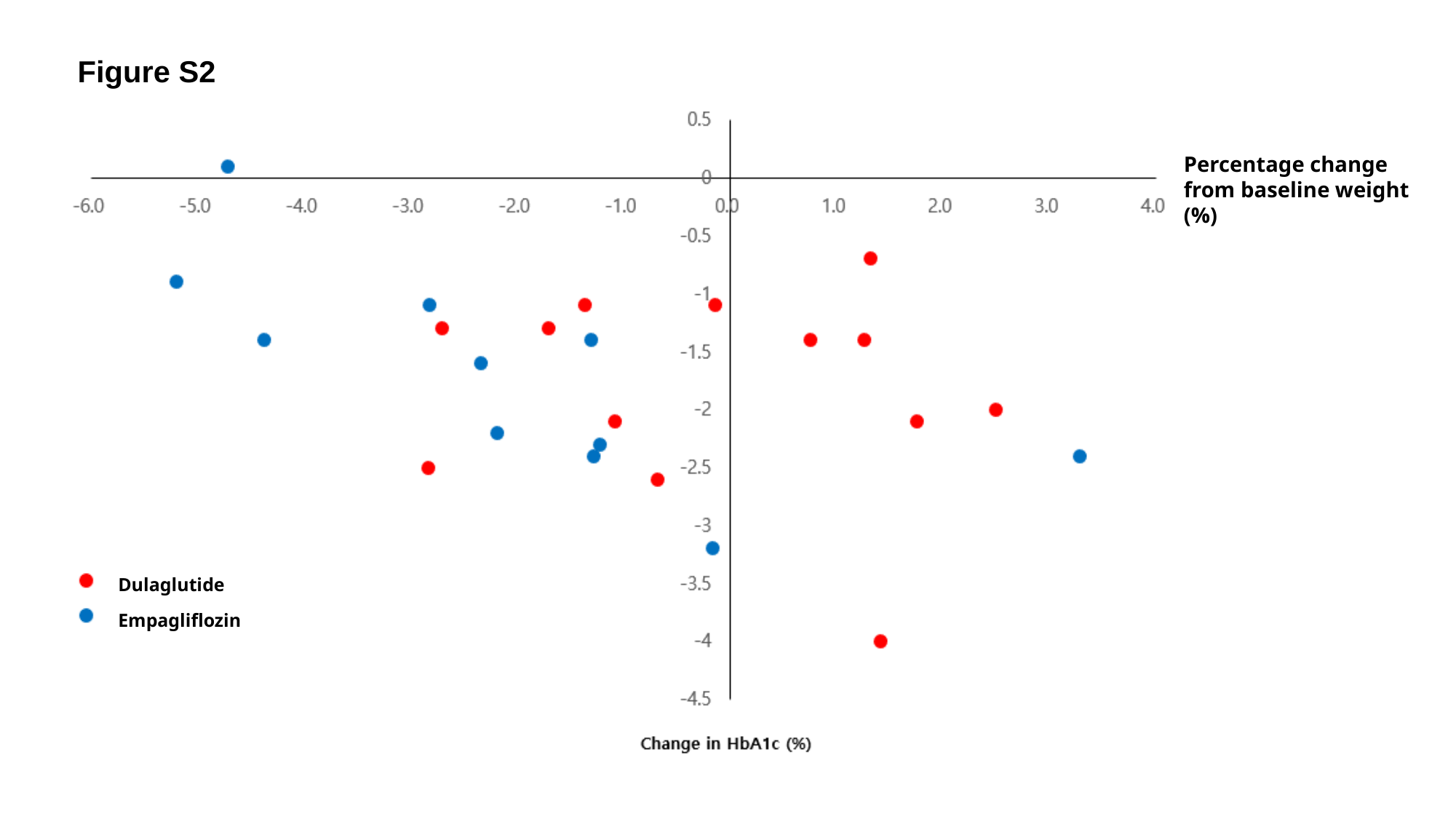

Figure S2
Dulaglutide
Empagliflozin
Percentage change from baseline weight (%)
Absolute change from baseline HbA1c (%)

## Slide 4
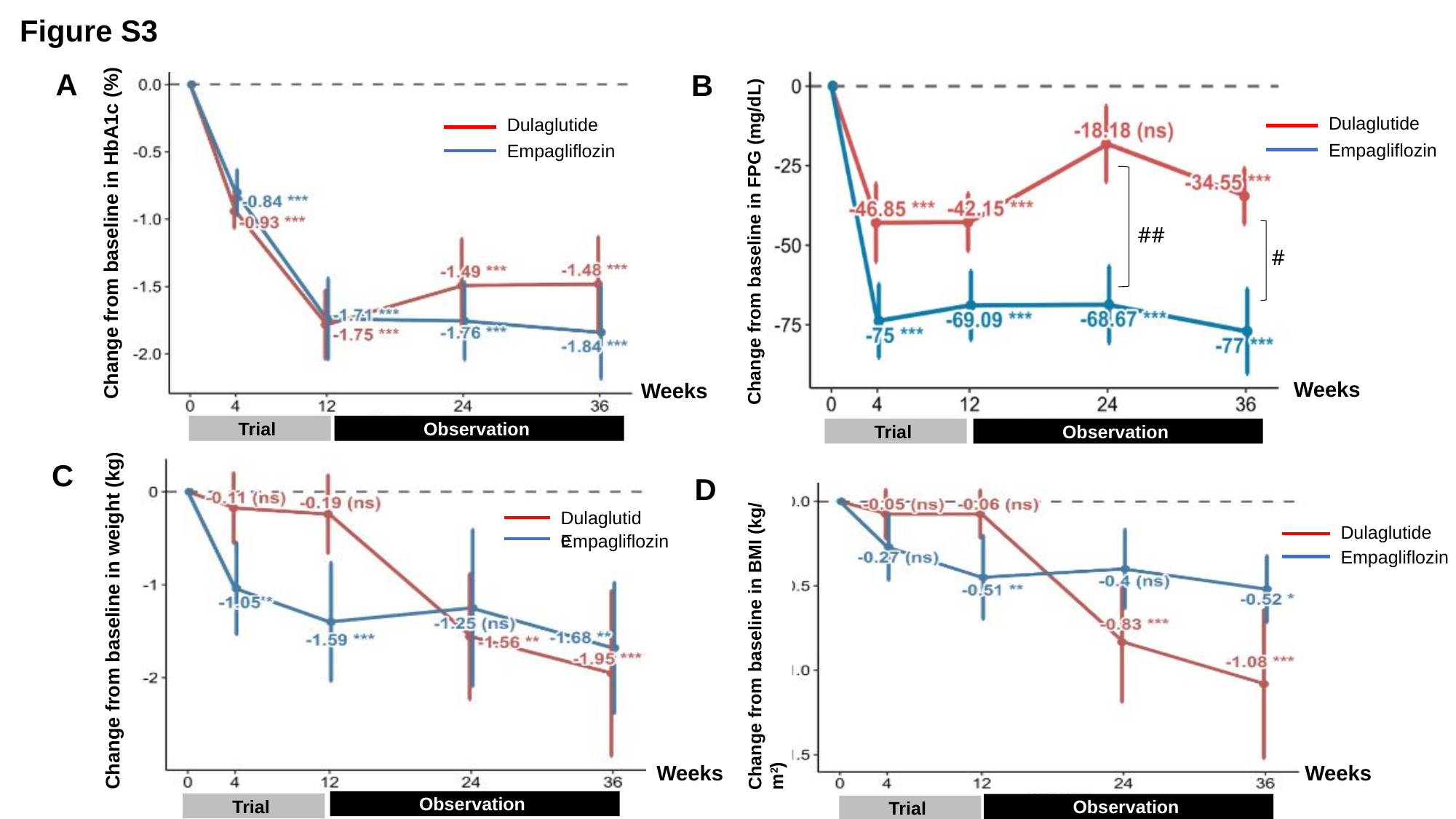

B
Dulaglutide
Empagliflozin
##
#
Weeks
Trial
Observation
Change from baseline in FPG (mg/dL)
Figure S3
Change from baseline in HbA1c (%)
A
Dulaglutide
Empagliflozin
Weeks
Trial
Observation
C
Change from baseline in weight (kg)
Weeks
Observation
Trial
D
Change from baseline in BMI (kg/m2)
Weeks
Observation
Trial
Dulaglutide
Empagliflozin
Dulaglutide
Empagliflozin
Dulaglutide
Empagliflozin
Dulaglutide
Empagliflozin

## Slide 5
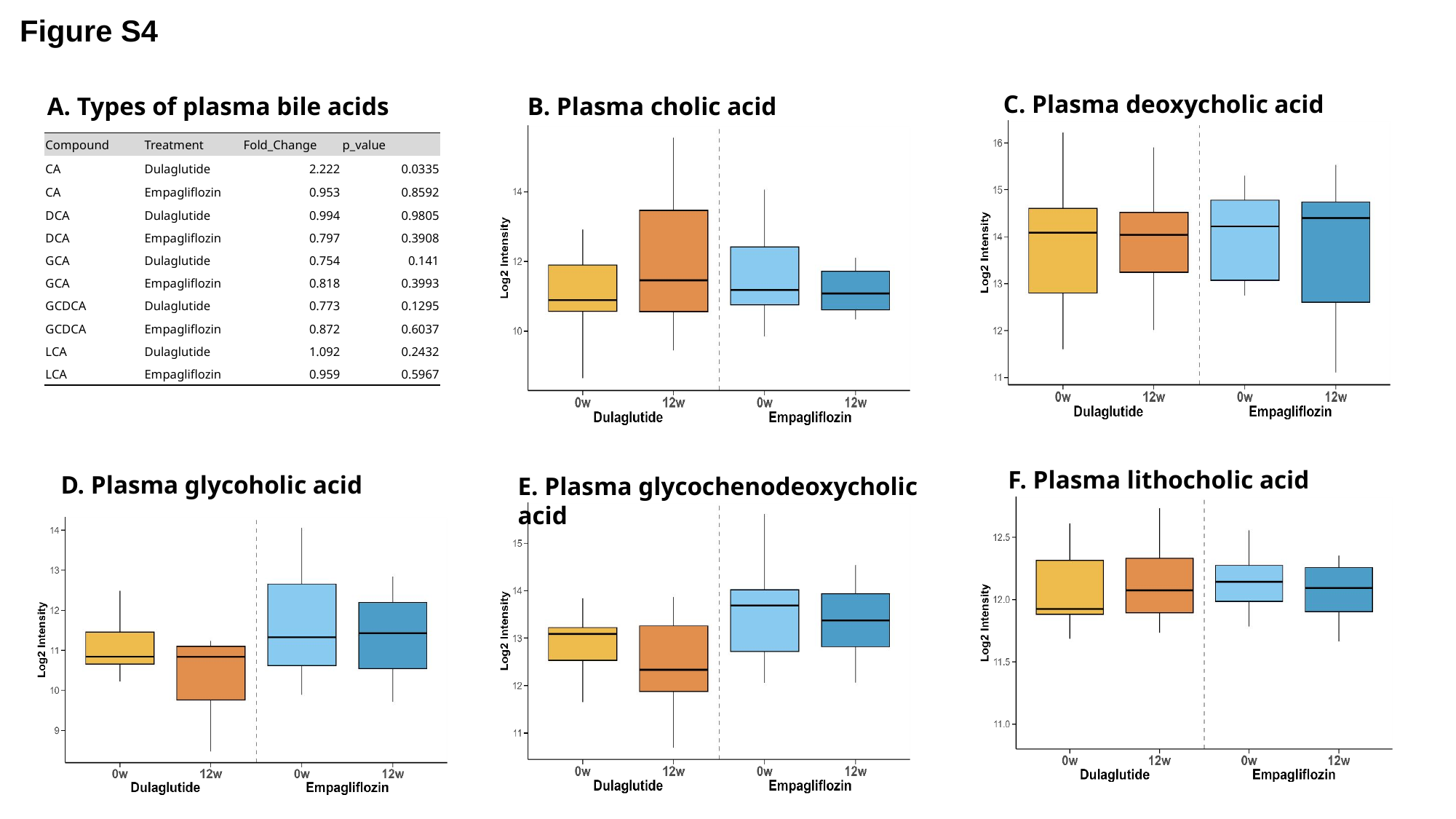

Figure S4
C. Plasma deoxycholic acid
A. Types of plasma bile acids
B. Plasma cholic acid
| Compound | Treatment | Fold\_Change | p\_value |
| --- | --- | --- | --- |
| CA | Dulaglutide | 2.222 | 0.0335 |
| CA | Empagliflozin | 0.953 | 0.8592 |
| DCA | Dulaglutide | 0.994 | 0.9805 |
| DCA | Empagliflozin | 0.797 | 0.3908 |
| GCA | Dulaglutide | 0.754 | 0.141 |
| GCA | Empagliflozin | 0.818 | 0.3993 |
| GCDCA | Dulaglutide | 0.773 | 0.1295 |
| GCDCA | Empagliflozin | 0.872 | 0.6037 |
| LCA | Dulaglutide | 1.092 | 0.2432 |
| LCA | Empagliflozin | 0.959 | 0.5967 |
F. Plasma lithocholic acid
D. Plasma glycoholic acid
E. Plasma glycochenodeoxycholic acid

## Slide 6
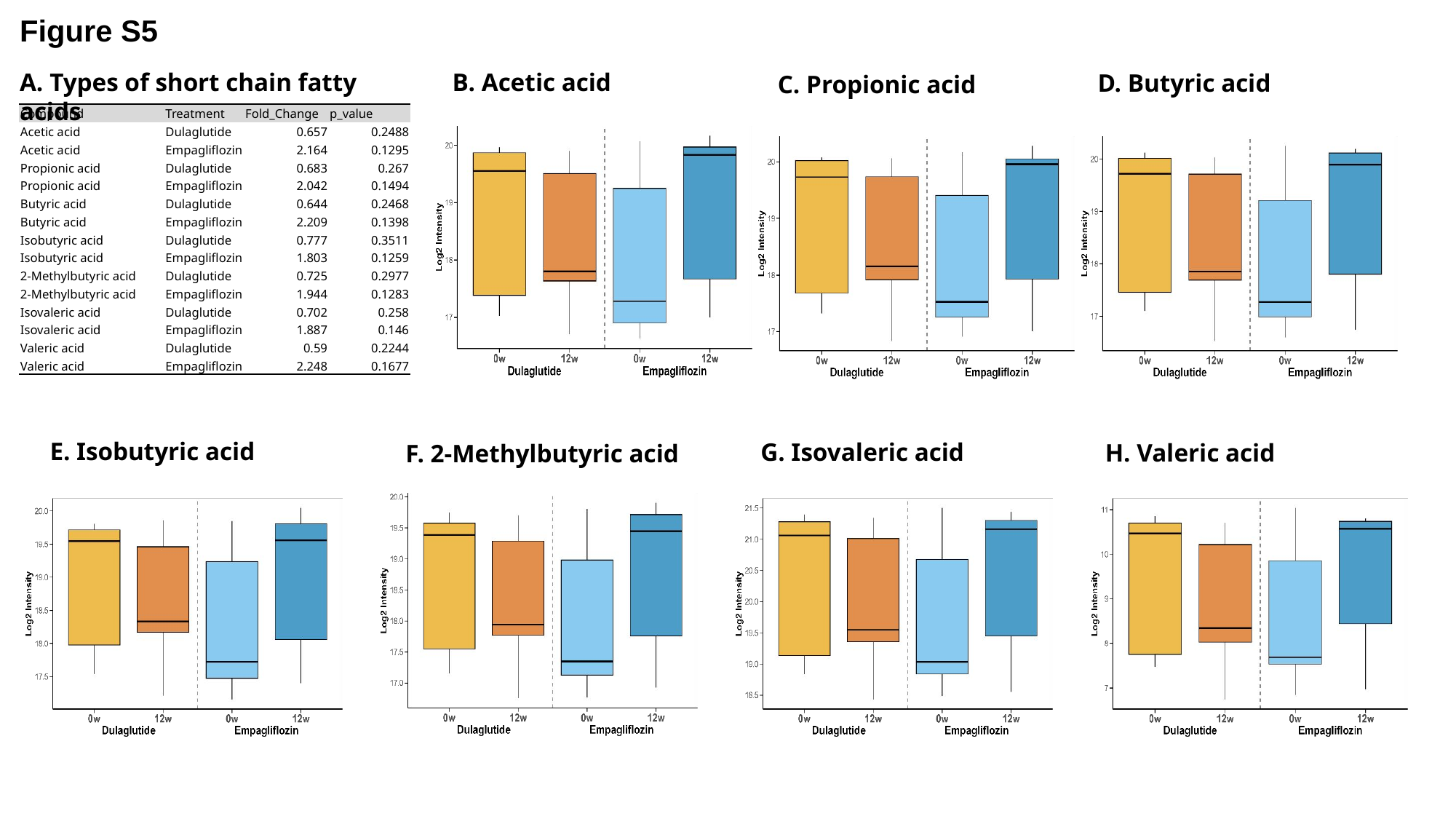

Figure S5
B. Acetic acid
A. Types of short chain fatty acids
D. Butyric acid
C. Propionic acid
| Compound | Treatment | Fold\_Change | p\_value |
| --- | --- | --- | --- |
| Acetic acid | Dulaglutide | 0.657 | 0.2488 |
| Acetic acid | Empagliflozin | 2.164 | 0.1295 |
| Propionic acid | Dulaglutide | 0.683 | 0.267 |
| Propionic acid | Empagliflozin | 2.042 | 0.1494 |
| Butyric acid | Dulaglutide | 0.644 | 0.2468 |
| Butyric acid | Empagliflozin | 2.209 | 0.1398 |
| Isobutyric acid | Dulaglutide | 0.777 | 0.3511 |
| Isobutyric acid | Empagliflozin | 1.803 | 0.1259 |
| 2-Methylbutyric acid | Dulaglutide | 0.725 | 0.2977 |
| 2-Methylbutyric acid | Empagliflozin | 1.944 | 0.1283 |
| Isovaleric acid | Dulaglutide | 0.702 | 0.258 |
| Isovaleric acid | Empagliflozin | 1.887 | 0.146 |
| Valeric acid | Dulaglutide | 0.59 | 0.2244 |
| Valeric acid | Empagliflozin | 2.248 | 0.1677 |
E. Isobutyric acid
G. Isovaleric acid
H. Valeric acid
F. 2-Methylbutyric acid

## Slide 7
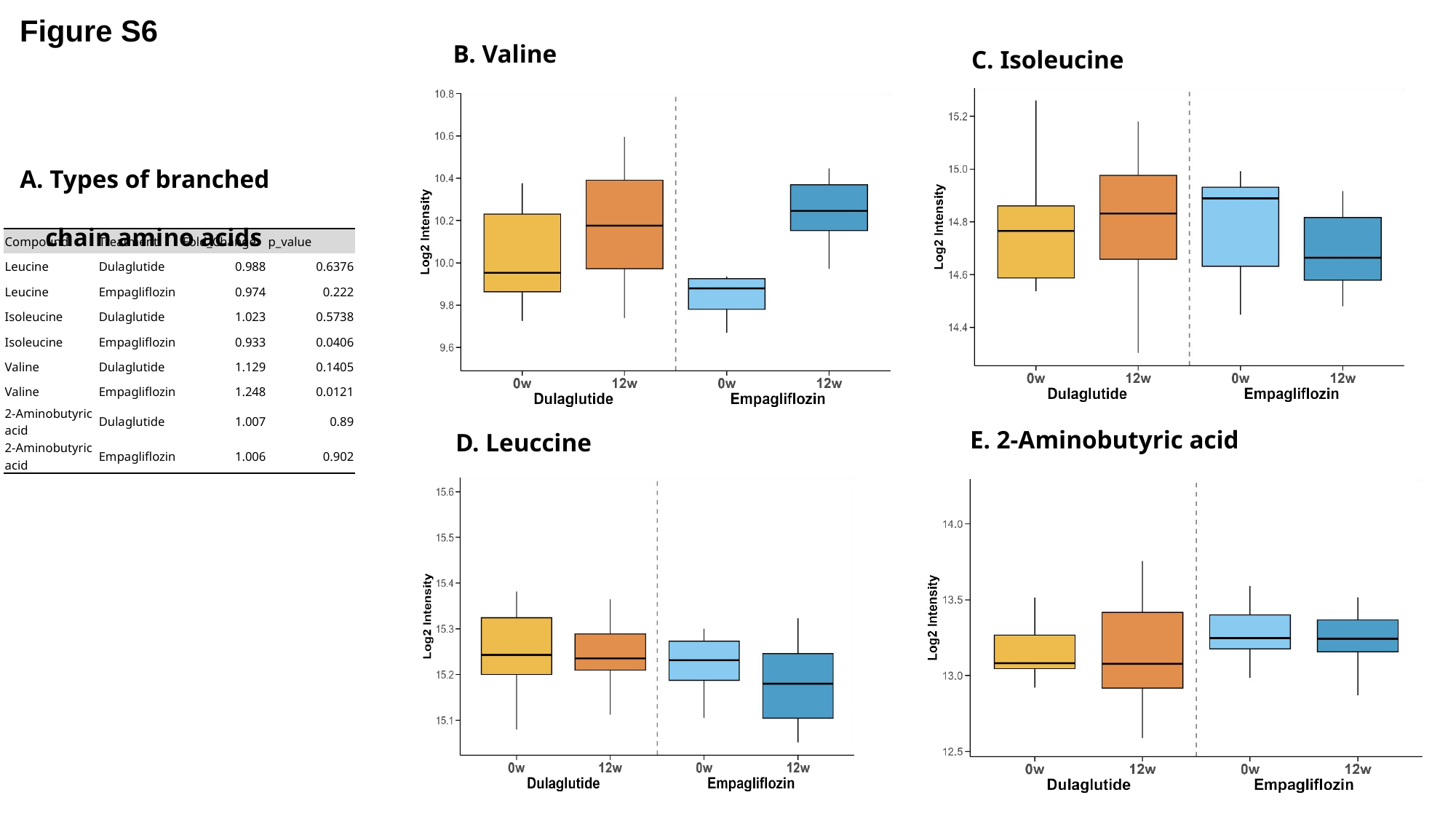

Figure S6
B. Valine
C. Isoleucine
A. Types of branched
 chain amino acids
| Compound | Treatment | Fold\_Change | p\_value |
| --- | --- | --- | --- |
| Leucine | Dulaglutide | 0.988 | 0.6376 |
| Leucine | Empagliflozin | 0.974 | 0.222 |
| Isoleucine | Dulaglutide | 1.023 | 0.5738 |
| Isoleucine | Empagliflozin | 0.933 | 0.0406 |
| Valine | Dulaglutide | 1.129 | 0.1405 |
| Valine | Empagliflozin | 1.248 | 0.0121 |
| 2-Aminobutyric acid | Dulaglutide | 1.007 | 0.89 |
| 2-Aminobutyric acid | Empagliflozin | 1.006 | 0.902 |
E. 2-Aminobutyric acid
D. Leuccine

## Slide 8
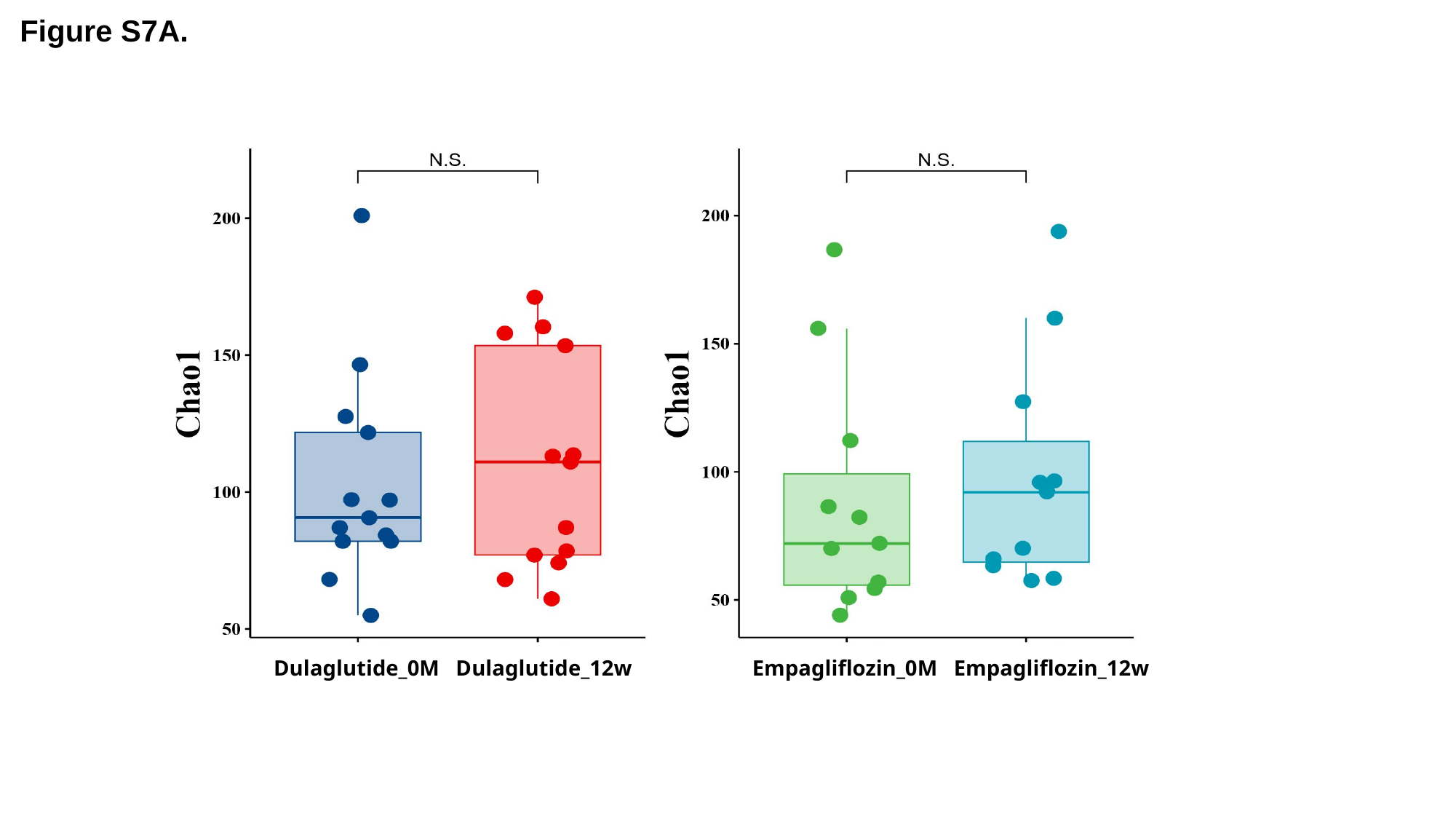

Figure S7A.
Dulaglutide_0M Dulaglutide_12w
Empagliflozin_0M Empagliflozin_12w

## Slide 9
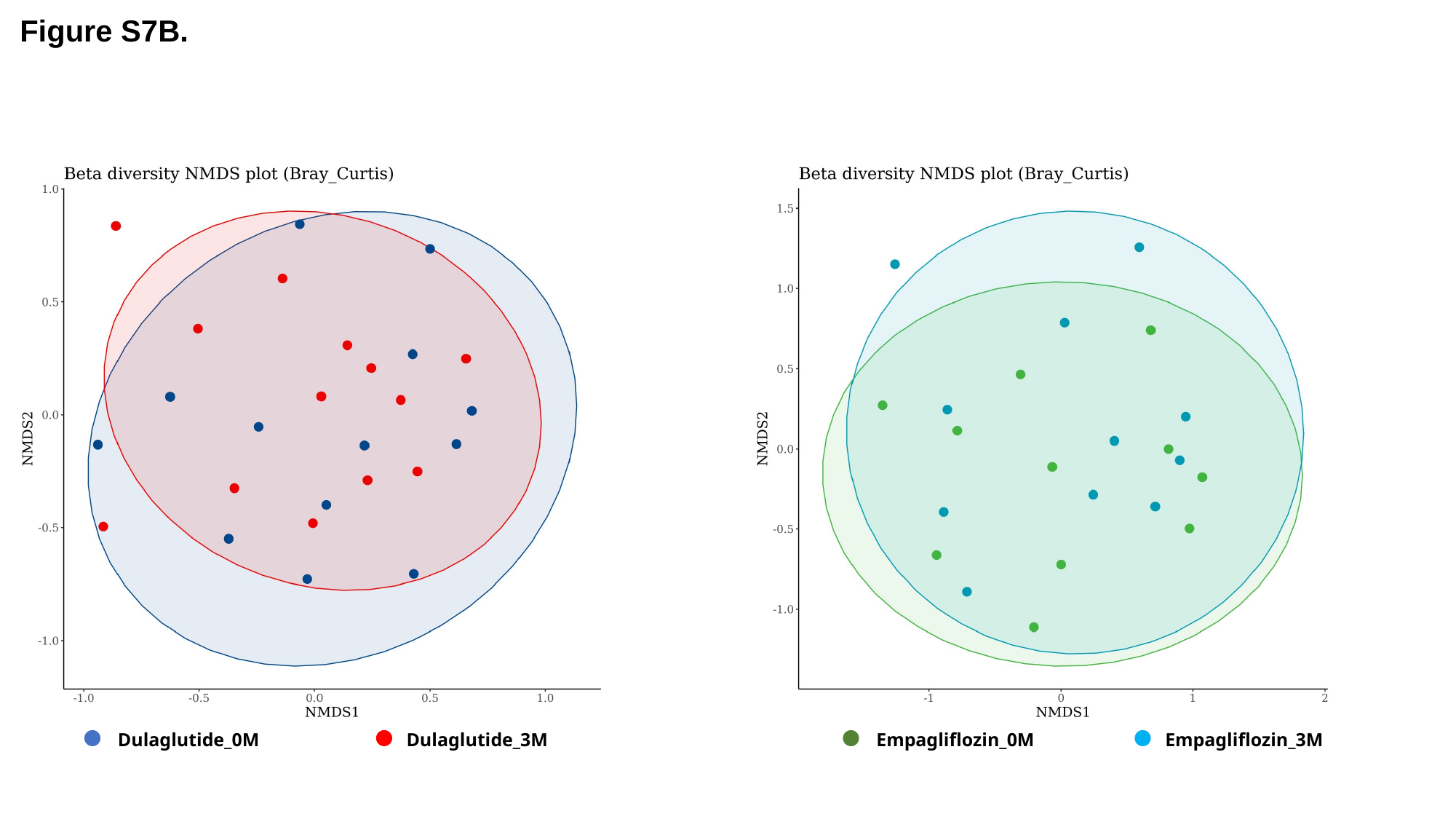

Figure S7B.
Empagliflozin_0M
Empagliflozin_3M
Dulaglutide_0M
Dulaglutide_3M
