# Supplementary material for: Dulaglutide versus empagliflozin as add-on therapy to metformin and sulfonylurea in type 2 diabetes: a randomized pilot study with exploratory metabolomic and microbiome analyses
Source: Front Endocrinol (Lausanne). 2026 Jun 17;17:1843595. doi: 10.3389/fendo.2026.1843595 (PMC13318618; doi:10.3389/fendo.2026.1843595)
Supplement: Supplementary file 2 [file DataSheet1.pdf]

Table S1. Changes in glycemic and anthropometric parameters during the post-trial extension period (weeks 24 and 36)

	**Dulaglutide (N = 12)**	**Empagliflozin (N = 10)**
**Week 24 (post-trial 3 months)**		
**HbA1c, %**		
Change from baseline, LS mean ± SE	-1.49 ± 0.23	-1.76 ± 0.26
LS mean difference [95% CI]	-0.26 ± 0.35 [-0.96, 0.43]	
P for comparing two groups	0.453	
P for comparing baseline	<0.001	<0.001
P for comparing week 12	0.206	0.503
**Fasting plasma glucose, mg/dL**		
Change from baseline, LS mean ± SE	-18.18 ± 9.35	-68.67 ± 10.30
LS mean difference [95% CI]	-50.50 ± 13.90 [-78.30, -22.70]	
P for comparing two groups	0.001	
P for comparing baseline	0.056	<0.001
P for comparing week 12	0.002	0.966
**Body weight, kg**		
Change from baseline, LS mean ± SE	-1.56 ± 0.55	-1.25 ± 0.67
LS mean difference [95% CI]	0.31 ± 0.87 [-1.42, 2.03]	
P for comparing two groups	0.723	
P for comparing baseline	0.006	0.066
P for comparing week 12	0.019	0.603
**BMI, kg/m^2**		
Change from baseline, LS mean ± SE	-0.83 ± 0.24	-0.40 ± 0.29
LS mean difference [95% CI]	0.43 ± 0.38 [-0.32, 1.18]	
P for comparing two groups	0.253	
P for comparing baseline	<0.001	0.174
P for comparing week 12	0.002	0.522
**Week 36 (post-trial 6 months)**		
**HbA1c, %**		
Change from baseline, LS mean ± SE	-1.48 ± 0.24	-1.84 ± 0.25
LS mean difference [95% CI]	-0.36 ± 0.35 [-1.05, 0.34]	
P for comparing two groups	0.307	
P for comparing baseline	<0.001	<0.001
P for comparing week 12	0.108	0.667

**Fasting plasma glucose, mg/dL**		
Change from baseline, LS mean ± SE	-34.55 ± 11.60	-77.00 ± 12.10
LS mean difference [95% CI]	-42.45 ± 16.80 [-75.86, -9.05]	
P for comparing two groups	0.013	
P for comparing baseline	0.004	<0.001
P for comparing week 12	0.458	0.507
**Body weight, kg**		
Change from baseline, LS mean ± SE	-1.95 ± 0.55	-1.68 ± 0.58
LS mean difference [95% CI]	0.27 ± 0.80 [-1.33, 1.88]	
P for comparing two groups	0.734	
P for comparing baseline	<0.001	0.005
P for comparing week 12	<0.001	0.632
**BMI, kg/m^2**		
Change from baseline, LS mean ± SE	-1.08 ± 0.24	-0.52 ± 0.26
LS mean difference [95% CI]	0.56 ± 0.35 [-0.14, 1.27]	
P for comparing two groups	0.116	
P for comparing baseline	<0.001	0.046
P for comparing week 12	<0.001	0.789

Table S2. Differentially abundant gut microbial taxa identified by LEfSe analysis after dulaglutide or empagliflozin treatment

Time	Feature	LDA	P value
**Dulaglutide**			
Baseline	Bacillus	3.08	0.039
	Sporosarcina	4.59	0.016
	Psychorobacillus	4.04	0.003
At 24 week	Pseudomonas	3.75	0.015
	Bacillaceae incertae Sedis	3.51	0.015
	Clostridium	3.08	0.049
	Tissierella sp.	3.03	0.015
	Alkaliphilus	2.58	0.034
**Empagliflozin**			
Baseline	Leuconostoc mesenterioides	3.96	0.040
At 24 week	Ligilactobacillus sp.	2.21	0.032

LDA, Linear Discriminant Analysis; LEfSe, Linear Discriminant Analysis Effect Size

Table S3. Safety outcomes during the 12-week treatment period

	**Dulaglutide (N = 13)**	**Empagliflozin (N = 12)**
Any TEAEs	3	3
Symptomatic hypoglycemia	1	2
Arthralgia	0	0
Hypersensitivity	0	0
Pancreatitis, acute	0	0
Elevated liver enzyme	0	0
Urinary tract infection	0	0
Genital infection	0	1
Nausea	1	0
Diarrhea	0	0
Vomiting	0	0
Constipation	1	0
Any TEAE related to study drug	0	0
Any TESAEs	0	0
Any TESAE related to study drug	0	0
Any TEAEs leading to discontinuation	0	0

TEAE: treatment-emergent adverse event.
TESAE, treatment-emergent serious adverse event.

Table S4. Changes in renal function (eGFR) during the study period

Parameters	Dulaglutide (N = 13)	Empagliflozin (N = 12)	P value
Baseline eGFR, mL/min/1.73m^2	95.9 ± 23.0	91.7 ± 16.9	0.613
eGFR at 12 weeks	93.2 ± 20.8	94.5 ± 18.1	0.874
ΔeGFR (12 weeks)	-2.7 ± 9.3	2.8 ± 12.3	0.240
eGFR at 36 weeks*	107.4 ± 32.8	99.2 ± 18.1	0.633
ΔeGFR (36 weeks)*	4.1 ± 13.1	5.8 ± 9.5	0.815

*Data at 36 weeks were available in a subset of participants (n = 5 in the dulaglutide group and n = 7 in the empagliflozin group).
